# Active Endothelial Inactivation of Hyperpermeability: The Role of Nitric Oxide-Driven cAMP/Epac1 Signaling

**DOI:** 10.3390/jcdd12090361

**Published:** 2025-09-17

**Authors:** Mauricio A. Lillo, Pía C. Burboa, Walter N. Durán

**Affiliations:** Department of Pharmacology, Physiology and Neuroscience, Rutgers-New Jersey Medical School, Newark, NJ 07103, USA; pb588@njms.rutgers.edu (P.C.B.); duran@njms.rutgers.edu (W.N.D.)

**Keywords:** endothelial hyperpermeability, cAMP/Epac1 signaling, eNOS localization, VASP phosphorylation

## Abstract

Endothelial hyperpermeability is a hallmark of diverse inflammatory and vascular pathologies, including sepsis, acute respiratory distress syndrome (ARDS), ischemia–reperfusion injury, and atherosclerosis. Traditionally considered a passive return to baseline following stimulus withdrawal, barrier recovery is now recognized as an active, endothelial-driven process. Earlier work identified individual components of this restorative phase, such as cyclic adenosine monophosphate (cAMP)/exchange protein directly activated by cAMP 1 (Epac1) signaling, Rap1/Rac1 activation, vasodilator-stimulated phosphoprotein (VASP) phosphorylation, and targeted cytoskeletal remodeling, as well as kinase pathways involving PKA, PKG, and Src. However, these were often regarded as discrete events lacking a unifying framework. Recent integrative analyses, combining mechanistic insights from multiple groups, reveal that nitric oxide (NO) generated early during hyperpermeability can initiate a delayed cAMP/Epac1 cascade. This axis coordinates Rap1/Rac1-mediated cortical actin polymerization, VASP-driven junctional anchoring, retro-translocation of endothelial nitric oxide synthase (eNOS) to caveolar domains, PP2A-dependent suppression of actomyosin tension, and Krüppel-like factor 2 (KLF2)-driven transcriptional programs that sustain endothelial quiescence. Together, these pathways form a temporally orchestrated, multi-tiered “inactivation” program capable of restoring barrier integrity even in the continued presence of inflammatory stimuli. This conceptual shift reframes NO from solely a barrier-disruptive mediator to the initiating trigger of a coordinated, pro-resolution mechanism. The unified framework integrates cytoskeletal dynamics, junctional reassembly, focal adhesion turnover, and redox/transcriptional control, providing multiple potential intervention points. Therapeutically, Epac1 activation, Rap1/Rac1 enhancement, RhoA/ROCK inhibition, PP2A activation, and KLF2 induction represent strategies to accelerate endothelial sealing in acute microvascular syndromes. Moreover, applying these mechanisms to arterial endothelium could limit low-density lipoprotein (LDL) entry and foam cell formation, offering a novel adjunctive approach for atherosclerosis prevention. In this review, we will discuss both the current understanding of endothelial hyperpermeability mechanisms and the emerging pathways of its active inactivation, integrating molecular, structural, and translational perspectives.

## 1. Introduction

The vascular endothelium is a dynamic monolayer of endothelial cells lining the interior surface of blood vessels, serving as a critical barrier that regulates the exchange of fluids, solutes, and cells between the bloodstream and surrounding tissues. This selective permeability is essential for maintaining tissue homeostasis and responding to physiological stimuli. Disruption of this barrier function, termed endothelial hyperpermeability, is a hallmark of various pathological conditions, including sepsis, acute respiratory distress syndrome (ARDS), and ischemia–reperfusion injury. In these conditions, increased vascular permeability leads to tissue edema, inflammation, and organ dysfunction, contributing significantly to morbidity and mortality [1,2].

Endothelial activation and dysfunction are now recognized as pivotal contributors to barrier compromise across vascular beds, and they play a central role in the pathogenesis of inflammatory and cardiovascular diseases [3]. In particular, endothelial cell (EC) activation leads to structural and biochemical changes—including junctional protein phosphorylation, cytoskeletal remodeling, and altered gene expression—that facilitate vascular leakage. While prior studies have described the involvement of kinase-dependent pathways in barrier recovery [4,5,6]. Our review highlights a novel initiating signal in this process: nitric oxide (NO). We propose that NO, generated early during hyperpermeability, triggers a cAMP/Epac1-mediated program that actively restores barrier integrity. This concept reframes barrier recovery not as a passive closure phase, but as a NO-driven endothelial self-repair mechanism.

Traditionally, the resolution of endothelial hyperpermeability was considered a passive process, occurring upon the removal of inflammatory stimuli. However, emerging evidence suggests that endothelial cells possess intrinsic mechanisms to actively restore barrier integrity [7], even amidst ongoing inflammatory challenges. This paradigm shift underscores the endothelium’s active role in maintaining vascular homeostasis and its potential as a therapeutic target [8,9,10,11,12,13,14,15,16,17].

The regulation of endothelial permeability includes complex signaling pathways and structural components that govern intercellular junctions and cytoskeletal dynamics. Key among these are the tight junctions and adherens junctions, which are composed of transmembrane proteins such as occludin, claudin, and vascular endothelial (VE)-cadherin. These junctional complexes are connected to the actin cytoskeleton and are modulated by various signaling molecules, including small GTPases, such as RhoA and Rac1, which influence cytoskeletal tension and cell contraction. Disruption of these junctions, often mediated by inflammatory cytokines and vasoactive agents, leads to increased paracellular permeability [4,18,19,20].

One of the pivotal signaling molecules in this context is endothelial nitric oxide synthase (eNOS), which produces nitric oxide (NO), a vasodilator that also modulates vascular permeability. The subcellular localization of eNOS—whether at the plasma membrane, cytosol, or Golgi apparatus—significantly influences its activity and, consequently, endothelial function. Inflammatory stimuli induce the translocation of eNOS from the plasma membrane to the cytosol, enhancing NO production and promoting hyperpermeability [16,17,21]. Conversely, the return of eNOS to the plasma membrane is associated with the restoration of barrier integrity [15,22,23].

The cyclic adenosine monophosphate (cAMP)/exchange protein directly activated by cAMP 1 (Epac1) signaling pathway has emerged as a crucial regulator of endothelial barrier function. Activation of Epac1 leads to the strengthening of intercellular junctions and stabilization of the cytoskeleton, thereby reducing permeability. Additionally, Epac1 activation promotes the phosphorylation of vasodilator-stimulated phosphoprotein (VASP), an actin-binding protein that facilitates cytoskeletal rearrangement and enhances barrier integrity [24,25,26,27].

Understanding these intricate mechanisms offers potential therapeutic avenues for diseases characterized by vascular barrier dysfunction. Targeting the cAMP/Epac1 signaling axis, modulating eNOS localization, and enhancing VASP activity could provide novel strategies to reinforce endothelial barrier function in inflammatory diseases. Further research into these pathways may lead to the development of targeted therapies aimed at mitigating endothelial hyperpermeability and associated pathologies.

## 2. Mechanisms Underlying Endothelial Hyperpermeability

Endothelial hyperpermeability is a hallmark of various pathological conditions, including sepsis, acute respiratory distress syndrome (ARDS), and ischemia–reperfusion injury. This phenomenon arises from the disassembly of intercellular junctions and cytoskeletal rearrangements, leading to increased paracellular gaps. Several key mediators contribute to this process, notably inflammatory cytokines, vasoactive agents, reactive oxygen species (ROS), and vascular endothelial growth factor (VEGF) [2].

### 2.1. Inflammatory Cytokines

Pro-inflammatory cytokines, notably tumor necrosis factor-alpha (TNF-α) and interleukin-1β (IL-1β), are key mediators of endothelial barrier disruption across vascular beds, including the systemic microcirculation and the blood–brain barrier. TNF-α engages TNFR1/2 receptors to activate multiple signaling cascades, among which the ERK1/2–MLCK axis is critical. ERK1/2-dependent phosphorylation of myosin light chain kinase (MLCK) enhances myosin light chain (MLC) phosphorylation, triggering actomyosin contraction, stress fiber formation, and disassembly of tight and adherens junctions [28]. This contractile response promotes paracellular gap formation, thereby increasing permeability and enabling plasma protein leakage and leukocyte transmigration into perivascular tissues. In parallel, TNF-α downregulates the transcription and membrane localization of tight junction components such as occludin, claudin-5, and ZO-1, destabilizing the junctional complex and exacerbating barrier dysfunction [28].

Similarly, IL-1β, via IL-1R1–MyD88 signaling, activates p38 MAPK and ERK1/2, converging on cytoskeletal remodeling and tight junction disassembly. This cytokine also engages RhoA/ROCK-mediated pathways, further amplifying MLC phosphorylation and endothelial contraction. Both TNF-α and IL-1β enhance Ca^2+^ influx through store-operated and receptor-operated channels, with subsequent Ca^2+^/calmodulin activation of MLCK, reinforcing the contractile and barrier-disruptive phenotype. These effects are not limited to paracellular permeability: increased vesicular trafficking via caveolae-mediated transcytosis has been reported, contributing to macromolecular leakage independent of junctional gaps [29].

In the context of the blood–brain barrier, these cytokines induce reorganization of junctional complexes, pericyte detachment, and astrocytic endfoot alterations, thereby weakening barrier selectivity and facilitating neuroinflammatory responses. The temporal dynamics of these signaling events suggest that acute TNF-α/IL-1β exposure drives a rapid, active barrier breakdown through Ca^2+^/calmodulin and RhoA/ROCK-dependent mechanisms, followed by transcriptional repression of junctional proteins that sustains hyperpermeability during chronic inflammation [30].

Importantly, while these cytokines are potent initiators of barrier breakdown, several studies indicate that endothelial cells possess intrinsic mechanisms to actively restore barrier integrity once the inflammatory stimulus subsides [31]. This involves reassembly of tight and adherens junctions, actin cytoskeleton relaxation, and upregulation of junctional proteins via cAMP–Epac–Rap1, Rac1, and PI3K/Akt pathways [32]. Understanding the molecular switches that transition the endothelium from a barrier-disruptive to a barrier-restorative state is crucial, as pharmacological targeting of these recovery mechanisms may represent a viable strategy to limit tissue injury and accelerate resolution in inflammatory diseases.

### 2.2. Vasoactive Agents

Vasoactive substances, such as histamine and thrombin, are potent, rapid-acting mediators of endothelial hyperpermeability whose effects are largely mediated through cytoskeletal contraction and junctional remodeling. Histamine binds to G protein–coupled H1 receptors on endothelial cells, triggering Gq/11-mediated activation of phospholipase Cβ (PLCβ), which catalyzes the hydrolysis of phosphatidylinositol 4,5-bisphosphate into diacylglycerol and inositol 1,4,5-trisphosphate (IP_3_). IP_3_ promotes Ca^2+^ release from the endoplasmic reticulum via IP_3_ receptors, while diacylglycerol activates protein kinase C (PKC). The rise in cytosolic Ca^2+^ activates calmodulin-dependent myosin light chain kinase (MLCK), leading to phosphorylation of myosin light chains (MLC) and actomyosin contraction. In parallel, histamine engages RhoA/Rho kinase (ROCK) signaling, which inhibits myosin phosphatase through phosphorylation of its myosin-binding subunit (MYPT1), thereby sustaining MLC phosphorylation and contractile tension. The combined effect is stress fiber formation, adherens junction (VE-cadherin) disassembly, and the opening of paracellular gaps [33,34,35].

Thrombin, via protease-activated receptor-1 (PAR-1) and PAR-4 activation, initiates a similar but more prolonged contractile response. Coupling to Gq/11 and G12/13 proteins leads to both PLCβ–Ca^2+^/calmodulin–MLCK activation and robust RhoA/ROCK-mediated inhibition of myosin phosphatase [35,36,37]. Thrombin additionally recruits Src family kinases and focal adhesion kinases (FAKs), promoting phosphorylation of junctional proteins and reorganization of focal adhesions. This dual cytoskeletal and junctional targeting facilitates both paracellular gap formation and enhanced transcellular vesicular transport [35,36,37]. Notably, the magnitude and duration of thrombin-induced hyperpermeability depend on endothelial cell type, confluency, and pre-existing inflammatory priming [35,36,37].

Emerging evidence further shows that these vasoactive mediators not only cause acute barrier breakdown but also activate early signals that prime the endothelium for recovery. For example, in models of histamine- or thrombin-induced permeability, transient activation of Rac1 and Rap1 downstream of cAMP–Epac signaling contributes to junctional reassembly within 30–60 min after the initial contractile phase [6,33,35,36,37]. This biphasic response—rapid contraction followed by intrinsic restoration—highlights a broader paradigm in endothelial biology: barrier-disruptive and barrier-restorative processes are not mutually exclusive but rather sequential and dynamically regulated. Understanding this temporal interplay offers a therapeutic opportunity to pharmacologically enhance endogenous restoration mechanisms while attenuating the initial contractile insult [33,37,38,39,40,41,42].

### 2.3. Reactive Oxygen Species

Oxidative stress, defined by the excessive production of reactive oxygen species (ROS) such as superoxide anion (O_2_•^−^), hydrogen peroxide (H_2_O_2_), and hydroxyl radicals, is a central mechanism in endothelial barrier dysfunction. Major sources of ROS in endothelial cells include NADPH oxidases (NOX2, NOX4), uncoupled endothelial nitric oxide synthase (eNOS), xanthine oxidase, and dysfunctional mitochondria [43,44]. Superoxide rapidly reacts with nitric oxide (NO) to generate peroxynitrite (ONOO^−^), which nitrates tyrosine residues in junctional and cytoskeletal proteins, altering their conformation and function [43,44].

ROS impair tight junction integrity through both direct oxidative modifications and activation of signaling pathways. Oxidative modifications—such as S-glutathionylation, carbonylation, and tyrosine nitration—affect occludin, claudin-5, and zonula occludens-1 (ZO-1), leading to their endocytosis and degradation [4,6,30,43,45]. ROS also activate matrix metalloproteinases (MMP-2, MMP-9), which proteolytically cleave VE-cadherin and other junctional proteins, weakening cell–cell adhesion [46].

At the signaling level, ROS engage redox-sensitive kinases such as Src family kinases, PKC, and MAPKs (ERK1/2, JNK, p38), which phosphorylate junctional proteins and actin regulators, promoting cytoskeletal contraction. ROS also activate RhoA/ROCK, leading to increased myosin light chain (MLC) phosphorylation and stress fiber formation [32,35,37,44]. In parallel, NF-κB activation drives the transcription of pro-inflammatory mediators, amplifying vascular leakage [6,35,43].

Mitochondrial ROS (mtROS) further contribute by oxidizing ryanodine and IP_3_ receptors, facilitating Ca^2+^ release from the endoplasmic reticulum, thereby activating Ca^2+^/calmodulin-dependent MLCK and increasing actomyosin contractility [3,46]. This interplay between oxidative stress, Ca^2+^ mobilization, and cytoskeletal remodeling is a critical amplifier of barrier breakdown in both acute and chronic vascular inflammation [20,47,48,49,50,51,52,53,54,55,56,57,58,59,60,61].

### 2.4. Vascular Endothelial Growth Factor (VEGF)

Vascular endothelial growth factor A (VEGF-A) is a prototypical inducer of endothelial hyperpermeability in addition to its role in angiogenesis. Upon binding and dimerizing vascular endothelial growth factor receptor-2 (VEGFR-2/KDR), the receptor undergoes autophosphorylation on key tyrosine residues that initiate parallel signaling cascades converging on junctional disassembly and cytoskeletal contraction [62,63,64,65]. Phosphorylation of Y1175 recruits phospholipase Cγ (PLCγ), generating IP_3_ and diacylglycerol (DAG), which, respectively, promote Ca^2+^ release via IP_3_ receptors and protein kinase C (PKC) activation. The resulting cytosolic Ca^2+^ elevation activates calmodulin-dependent myosin light chain kinase (MLCK) and, via the PI3K/Akt pathway, phosphorylates endothelial nitric oxide synthase (eNOS) at Ser1177, increasing nitric oxide (NO) production [62,63,64,65]. NO destabilizes adherens junctions (AJs) by promoting VE-cadherin/β-catenin dissociation and actomyosin-driven gap formation.

In parallel, VEGFR-2 engages Src family kinases through the Y949 (Y951 in human)–TSAd signaling axis, a pathway essential for permeability regulation in vivo [62,63,64,65]. Src-mediated phosphorylation of VE-cadherin recruits β-arrestin-2, triggering clathrin-mediated internalization of VE-cadherin and opening paracellular gaps [62,63,64,65]. VEGF also stimulates vesicular transport pathways, including caveolae and the vesiculo-vacuolar organelle (VVO), which provide a transcellular route for plasma proteins during hyperpermeability responses. In the central nervous system, VEGF decreases expression and membrane anchoring of tight junction (TJ) proteins such as claudin-5 and occludin, thereby weakening the blood–brain barrier (BBB) and contributing to neuroinflammation [62,63,64,65].

Finally, VEGF induces a rapid, spatially restricted burst of reactive oxygen species (ROS) through NADPH oxidases (NOX2, NOX4) and mitochondria. This ROS production forms a positive feedback loop that amplifies VEGFR-2/Src signaling, increases intracellular Ca^2+^, and further destabilizes junctional complexes [62,63,64,65]. Redox-dependent modifications, together with NO-mediated S-nitrosylation of junctional proteins, synergistically disrupt AJ and TJ integrity.

Collectively, VEGF integrates multiple pathways—PLCγ–Ca^2+^–MLCK, PI3K/Akt–eNOS–NO, VEGFR-2(Y949)–TSAd–Src–VE-cadherin endocytosis, NOX/mtROS production, and enhanced transcytosis via caveolae/VVO—to produce a potent and often biphasic hyperpermeability response [66,67,68,69,70,71,72,73,74,75,76,77,78]. This VEGF-driven network acts in concert with cytokines, vasoactive mediators, and oxidative stress to dismantle the endothelial barrier, highlighting multiple therapeutic targets, from Y949/Src blockade and VE-cadherin internalization inhibitors to antioxidant strategies and agents that enhance Epac–Rap1/Rac1-mediated barrier restoration [62,63,64,65,66,67,68,69,70,71,72,73,74,75,76,77,78].

## 3. eNOS Localization and Its Role in Hyperpermeability

Endothelial nitric oxide synthase is a pivotal enzyme in the regulation of vascular tone and permeability. Its subcellular localization significantly influences its activity and function.

### 3.1. Plasma Membrane

In resting endothelial cells, eNOS is predominantly localized in caveolae-rich domains of the plasma membrane. Within these microdomains, eNOS interacts with caveolin-1 (Cav-1), a scaffolding protein that inhibits eNOS activity under basal conditions. Upon stimulation by agonists such as acetylcholine, intracellular calcium levels rise, leading to the dissociation of eNOS from Cav-1 and subsequent activation of eNOS. This activation results in the production of nitric oxide, which diffuses to adjacent smooth muscle cells, causing vasodilation and contributing to vascular homeostasis. The localization of eNOS to caveolae is facilitated by post-translational modifications, including N-myristoylation and palmitoylation, which anchor the enzyme to the plasma membrane [79,80,81,82,83].

### 3.2. Cytosol

Upon stimulation by inflammatory agonists, eNOS translocates from the plasma membrane to the cytosol. This translocation is associated with increased NO production, leading to S-nitrosylation of junctional proteins and subsequent hyperpermeability. The presence of eNOS in the cytosol is necessary for PAF-induced hyperpermeability, highlighting the importance of eNOS localization in the regulation of endothelial barrier function. The internalization of eNOS via caveolae is a critical step in this process, as inhibition of caveolar endocytosis blocks eNOS translocation and attenuates hyperpermeability responses [14,16,17,21,84,85].

### 3.3. Golgi Apparatus

eNOS is found in the Golgi apparatus, where it undergoes post-translational modifications essential for its proper function and trafficking. The Golgi-associated eNOS is involved in the maturation and processing of the enzyme, preparing it for subsequent localization to the plasma membrane or other cellular compartments. Disruption of the Golgi function impairs eNOS activity and NO production, underscoring the significance of this organelle in eNOS regulation [82,83].

### 3.4. Dynamic Trafficking and Regulation

The dynamic trafficking of eNOS between the plasma membrane, cytosol, and Golgi apparatus is crucial for the regulation of endothelial barrier function. This trafficking is modulated by various factors, including post-translational modifications, protein–protein interactions, and cellular signaling pathways. For instance, the nitric-oxide synthase trafficking inducer (NOSTRIN) has been identified as a regulator of eNOS localization, sequestering eNOS and attenuating NO production. Additionally, the phosphorylation state of eNOS influences its subcellular distribution and activity, with specific phosphorylation sites promoting either membrane association or cytosolic localization [83,86,87,88,89].

### 3.5. Additional Mechanistic Pathways

Beyond the traditional pathways, emerging evidence suggests that other mechanisms may influence eNOS localization and function. For example, the interaction between eNOS and heat shock protein 90 (Hsp90) enhances eNOS activity and stabilizes its association with the plasma membrane. Furthermore, oxidative stress and reactive oxygen species can modulate eNOS localization and activity, potentially contributing to endothelial dysfunction and hyperpermeability. Understanding these additional pathways provides a more comprehensive view of the complex regulation of eNOS and its role in vascular permeability [86,90,91,92,93].

In summary, the subcellular localization of eNOS is a critical determinant of its activity and function in endothelial cells. The dynamic trafficking of eNOS between the plasma membrane, cytosol, and Golgi apparatus allows for precise regulation of NO production, which in turn influences vascular tone and permeability. Disruptions in eNOS localization and function can lead to pathological conditions characterized by increased vascular permeability, highlighting the importance of understanding the mechanisms governing eNOS trafficking and activity.

## 4. Active Inactivation of Hyperpermeability: A Paradigm Shift

Recent evidence has shifted the understanding of endothelial barrier recovery from a passive decay of hyperpermeability—thought to occur simply after withdrawal of inflammatory or mechanical stimuli—toward an active, highly orchestrated process. Although numerous kinases had previously been implicated in the recovery phase, and studies such as Aslam et al., 2014 [5] had already demonstrated that cAMP/Epac1 signaling contributes to barrier restoration, these observations were largely viewed as separate signaling events within the broader context of hyperpermeability. What had been missing was a unifying mechanistic link. Nepali et al., 2023 [7] provided this connection by demonstrating that nitric oxide (NO), generated during the early hyperpermeability phase, can trigger a delayed cAMP/Epac1 signaling cascade that actively promotes barrier restoration, even in the continued presence of the initiating stimulus. This concept reframes the role of NO from being solely a permeability-inducing mediator to serving as the initiating signal for a coordinated “inactivation” program. Importantly, this mechanistic framework integrates prior kinase observations into a single signaling sequence and opens new opportunities to identify therapeutic targets for a wide spectrum of conditions in which endothelial leak and barrier damage are central pathogenic features.

Consistent with this view, recent mechanistic analyses [6] demonstrate that endothelial barrier restoration is a highly orchestrated, multistage process rather than a passive return to baseline. The repair phase is initiated by sustained but spatially confined nitric oxide (NO) production, which, together with cAMP, activates the exchange protein directly activated by cAMP 1 (Epac1). This, in turn, triggers Rap1 and Rac1 activation, promoting cortical actin polymerization and the redistribution of junctional proteins such as VE-cadherin, β-catenin, and ZO-1 back to sites of cell–cell contact.

Importantly, this GTPase-driven remodeling is coupled to disassembly of central stress fibers and reorientation of actin filaments toward the cell periphery, relieving junctional tension and enabling zipper-like closure of intercellular gaps. Microtubule stabilization, mediated by CLIP-170 and IQGAP1 recruitment, further supports the directed trafficking of junctional components to the plasma membrane. Concurrently, Epac1–Rap1 signaling antagonizes RhoA/ROCK activity, preventing re-phosphorylation of myosin light chains and thereby reducing contractile forces that would otherwise re-open the barrier [6].

These findings position NO/cAMP/Epac1 not simply as permissive signals but as master coordinators of a pro-restorative program that integrates cytoskeletal dynamics, junctional reassembly, and focal adhesion turnover. This conceptual shift has translational implications: pharmacological activation of Epac1, selective enhancement of Rap1/Rac1 signaling, or targeted suppression of RhoA/ROCK during the repair phase could accelerate endothelial sealing in acute inflammatory settings such as sepsis, ischemia–reperfusion injury, or acute lung injury. Moreover, understanding how these pathways are temporally regulated opens the possibility of developing “pro-resolution” therapies that enhance endogenous restoration mechanisms without chronically suppressing physiological permeability required for immune surveillance and angiogenesis.

### 4.1. cAMP/Epac1 Signaling Axis

Central to this restorative program is the signaling axis formed by cyclic AMP (cAMP) and its downstream effector, exchange protein directly activated by cAMP 1 (Epac1). Inflammatory agonists such as platelet-activating factor (PAF) or histamine are classically recognized for their rapid, barrier-disruptive actions—inducing Ca^2+^ influx, cytoskeletal contraction, and junctional disassembly. However, several studies have shown that these same stimuli, when transient and not overwhelming, are followed by a delayed secondary rise in intracellular cAMP [4,5,7].This temporal shift in second messenger levels engages Epac1, initiating a switch from a contractile, barrier-compromised phenotype to a restorative, barrier-sealing state.

Epac1 activation exerts potent barrier-protective effects through multiple converging mechanisms [3,4,7,34,44,94]. First, it promotes cortical actin stabilization and reorganization of the cytoskeleton from centrally oriented stress fibers to a peripheral actin rim, reducing junctional tension and facilitating cell–cell adhesion [95,96]. Second, Epac1–Rap1 signaling drives the targeted trafficking and reassembly of adherens and tight junction proteins—VE-cadherin, β-catenin, ZO-1—back to intercellular junctions. This “zippering” effect is essential for closing paracellular gaps [6].

A particularly intriguing facet of Epac1 function is its ability to influence endothelial nitric oxide synthase (eNOS) localization. During the disruptive phase, inflammatory mediators promote eNOS displacement from the plasma membrane to the cytosol, favoring diffuse cytoplasmic NO production and increasing the likelihood of junctional protein S-nitrosylation, which destabilizes adhesive contacts [15]. Epac1 activation facilitates the re-localization of eNOS back to the plasma membrane, where NO production is spatially constrained and more effectively coupled to physiological vasodilatory signaling rather than barrier loosening [15]. By reducing cytosolic NO pools, Epac1 limits aberrant S-nitrosylation events and promotes the biochemical environment needed for stable junctional complexes.

Through these combined actions—cytoskeletal stabilization, junctional reassembly, and spatial control of NO signaling—Epac1 functions not just as a downstream mediator of cAMP, but as a master integrator that actively redefines endothelial phenotype from permeable to sealed. This dual role, following an initial inflammatory insult, underscores the potential for therapeutically targeting the cAMP–Epac1 axis to accelerate endogenous barrier restoration in acute vascular injury, sepsis, and ischemia–reperfusion syndromes.

### 4.2. VASP Phosphorylation and Cytoskeletal Stabilization

Epac1 activation is tightly linked to the phosphorylation of vasodilator-stimulated phosphoprotein (VASP), an actin-binding protein known to stabilize intercellular junctions. VASP is a substrate for protein kinase A (PKA) and protein kinase G (PKG), and its phosphorylation (particularly at Ser157 and Ser239) enhances its ability to facilitate actin filament elongation and junctional integrity [97]. Upon Epac1 activation, VASP migrates to the cortical actin zone and contributes to the re-anchoring of VE-cadherin and other adhesion molecules to the cytoskeleton, limiting paracellular leakage [98]. This pathway is essential not just for structural reinforcement but also for controlling signal transduction cascades linked to mechanical and chemical stress [97,99,100].

### 4.3. eNOS Relocation and Termination of the Permeability Response

A key insight from recent studies is that cytosolic eNOS, induced during early inflammation, is actively relocated back to membrane domains such as caveolae once Epac1/VASP signaling is engaged [7]. This retro-translocation significantly reduces cytosolic NO and the risk of aberrant S-nitrosylation of cytoskeletal and junctional proteins, a modification known to disrupt barrier architecture [15]. Membrane-localized eNOS, in contrast, is tightly regulated via interactions with caveolin-1 and Hsp90, allowing controlled NO release that supports, rather than undermines, endothelial integrity [7,14,15,90,101].

### 4.4. An Intrinsic Feedback Mechanism

Collectively, these coordinated responses constitute an intrinsic, temporally orchestrated feedback loop designed to resolve hyperpermeability even in the persistent presence of inflammatory triggers. This mechanism is independent of leukocyte clearance or cytokine withdrawal and instead reflects an endothelial self-programming capacity for homeostasis. The delay between initial hyperpermeability and restoration suggests the involvement of transcriptional regulation or epigenetic modification, although these remain speculative and under investigation.

### 4.5. Emerging Pathways in Inactivation

Beyond the canonical cAMP–Epac1 axis, emerging evidence identifies additional signaling nodes that reinforce the active inactivation of endothelial hyperpermeability. Downstream of Epac1, the small GTPase Rap1 strengthens adherens junctions by promoting VE-cadherin clustering at the plasma membrane and stabilizing its association with β-catenin and the cortical actin cytoskeleton, thereby limiting paracellular gap formation and accelerating barrier resealing during recovery [102,103,104]. Rap1 also engages Rac1, driving cortactin-dependent actin polymerization and recruitment of junctional scaffolding proteins, which enhances zipper-like closure of intercellular gaps.

Protein phosphatases, particularly protein phosphatase 2A (PP2A), play a pivotal role by tempering cytoskeletal contraction and facilitating junctional re-assembly [105]. In parallel, microtubule-stabilizing mechanisms involving CLIP-170 and IQGAP1 support targeted trafficking of adhesion complexes back to the plasma membrane, complementing actin-based sealing processes.

These post-translational events converge with transcriptional reprogramming pathways. Notably, Epac1 activation—together with hemodynamic cues—induces Krüppel-like factor 2 (KLF2), a master transcriptional regulator that promotes junctional protein synthesis, enhances antioxidant defenses, and suppresses NF-κB-driven inflammatory gene expression [94,106,107]. Given its role in upregulating antioxidant enzymes such as heme oxygenase-1 and superoxide dismutase, KLF2 may also mitigate oxidative stress during the recovery phase, preserving protein integrity and signaling competence.

Together, these interconnected pathways suggest that active inactivation is a multi-tiered program coupling rapid cytoskeletal and junctional remodeling with long-term transcriptional and redox homeostasis—ensuring not only the sealing of an acutely leaky barrier but also the maintenance of endothelial quiescence over time.

### 4.6. From Leaky Endothelium to Lipid-Laden Plaque: A Potential Macrovascular Application of Barrier Inactivation Mechanisms

Atherosclerosis is a progressive inflammatory disease of large- and medium-sized arteries in which the endothelium serves as the critical gatekeeper between circulating lipoproteins and the arterial wall [108,109]. Under physiological conditions, an intact endothelial barrier restricts the entry of low-density lipoprotein (LDL) particles into the subendothelial space. However, endothelial dysfunction—precipitated by hemodynamic stress, metabolic risk factors, or systemic inflammation—leads to increased junctional permeability, reduced nitric oxide bioavailability, and a shift toward a pro-adhesive, pro-inflammatory phenotype. These changes allow native LDL to infiltrate the intima, where it becomes trapped in the extracellular matrix and undergoes oxidative modification to oxidized LDL (oxLDL) [109].

OxLDL acts as both a chemotactic and activating signal for circulating monocytes, which adhere to the dysfunctional endothelium via upregulated ICAM-1 and VCAM-1, transmigrate into the intima, and differentiate into macrophages. Through scavenger receptors such as CD36 and SR-A, these macrophages internalize oxLDL and transform into lipid-engorged foam cells [109,110]. Foam cells release cytokines (e.g., TNF-α, IL-1β), growth factors, and matrix metalloproteinases, creating a self-reinforcing inflammatory loop that recruits additional immune cells, stimulates smooth muscle cell proliferation and migration, and progressively destabilizes the plaque’s fibrous cap. Over time, these processes underpin the growth, vulnerability, and potential rupture of the atherosclerotic plaque.

The molecular mechanisms described in this work—particularly nitric oxide (NO)-driven, cAMP/Epac1-mediated junctional reassembly, cytoskeletal stabilization, and targeted modulation of eNOS localization—may have translational relevance beyond microvascular inflammation. In theory, enhancing active inactivation of hyperpermeability in arterial endothelium could shorten the duration of LDL entry after transient disruptions, reduce subendothelial lipid retention, and slow foam cell accumulation in early lesions. While the primary focus of barrier repair research has been acute microvascular syndromes such as sepsis or ARDS, a strategic extension of these principles to macrovascular beds could complement established lipid-lowering and anti-inflammatory therapies. By preserving junctional integrity under atherogenic conditions, pharmacological activation of barrier restoration pathways might represent a novel adjunctive approach to reducing cardiovascular risk and delaying plaque progression [111,112,113].

### 4.7. Organ-Specific Endothelial Architecture and Responses to Injury

Endothelial structure is not uniform across vascular beds, and this heterogeneity has profound implications for barrier function, susceptibility to hyperpermeability, and the strategies of repair. In the brain, endothelial cells form the blood–brain barrier (BBB), where continuous tight and adherens junctions (claudin-5, occludin, ZO-1, VE-cadherin) restrict paracellular diffusion, while the lipid transporter MFSD2A represses caveolar transcytosis [114,115,116,117]. The barrier is further reinforced by pericyte coverage and astrocytic endfeet [114,117]. This highly restrictive organization minimizes basal leak but renders the BBB particularly vulnerable to abrupt junctional breakdown in response to cytokines, VEGF, or ischemia–reperfusion [114,115,117]. Under these conditions, permeability increases primarily through paracellular routes and reactivation of vesicular transport, facilitating neuroinflammation. Repair in the BBB therefore hinges on rapid “zippering” of tight junctions, suppression of transcytosis, and reconstitution of the perivascular support provided by astrocytes and pericytes [114,115,117]. Signaling pathways such as NO/cAMP/Epac1, Rap1/Rac1, and KLF2 are emerging as key coordinators of this reparative program.

By contrast, the renal microvasculature—especially glomerular capillaries—is adapted for high-volume filtration [118]. Endothelial cells here are fenestrated (~60–100 nm pores) with diaphragms formed by PV1/PLVAP and covered by a negatively charged glycocalyx that confers size- and charge-selectivity [119,120]. Unlike the BBB, where leak is primarily paracellular, renal permeability integrates both junctional complexes and fenestral pathways [118,119,120,121]. Injury in settings such as ischemia–reperfusion, diabetes, or sepsis is characterized by glycocalyx degradation, fenestra remodeling, increased expression of PLVAP, and junctional destabilization, resulting in proteinuria and albuminuria [118,119,120,121]. Recovery thus requires more than reassembly of tight and adherens junctions: restoration of glycocalyx composition, normalization of fenestral architecture, and rebalancing of VEGF-A/Ang-Tie2 signaling from podocytes are essential steps [118,119,120,121].

These organotypic contrasts illustrate that hyperpermeability is not a uniform event but instead reflects the baseline ultrastructure of each vascular bed. In the brain, barrier breakdown is catastrophic due to normally continuous junctions and suppressed transcytosis, while in the kidney, barrier dysfunction manifests as proteinuria due to combined glycocalyx loss, fenestral dysregulation, and junctional leak. Consequently, repair strategies—whether intrinsic, such as NO-driven cAMP/Epac1 signaling, or therapeutic—must be tailored to the structural context of each organ, emphasizing junctional reassembly in the BBB and combined glycocalyx–fenestra restoration in the glomerular endothelium.

## 5. Summary

**Figure 1:**
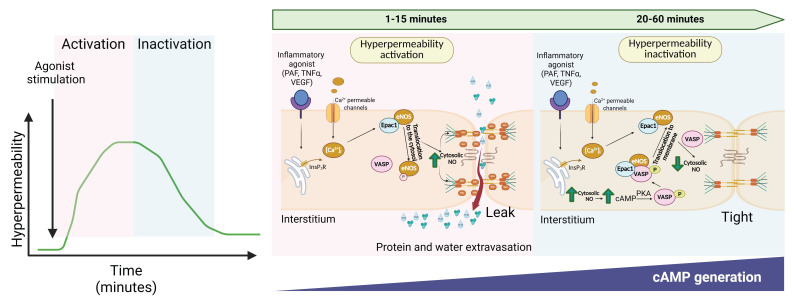
Mechanisms underlying the activation and active resolution of endothelial hyperpermeability. Temporal sequence of molecular events leading to endothelial hyperpermeability and its subsequent resolution. In the early phase (1–15 min), inflammatory agonists (PAF, TNF-α, VEGF) trigger Ca^2+^ influx and activation of cytosolic eNOS, resulting in nitric oxide (NO) production and S-nitrosylation of junctional and cytoskeletal proteins. These modifications compromise endothelial junctions, leading to barrier disruption and vascular leakage. During the recovery phase (20–60 min), a delayed increase in intracellular cAMP activates Epac1, which, in turn, stimulates Rap1 and promotes VASP phosphorylation. These events support cytoskeletal reorganization and VE-cadherin clustering. Concurrently, eNOS is retro-translocated to caveolar membrane domains where NO production is spatially restricted, mitigating S-nitrosylation-driven junctional disassembly. Collectively, this pathway highlights an intrinsic endothelial program that actively restores barrier integrity despite the continued presence of inflammatory stimuli.

Endothelial hyperpermeability is now understood as a highly orchestrated, active process rather than a passive decay following withdrawal of inflammatory or mechanical stimuli. Recent studies have unified previously disparate observations into a coherent mechanistic framework in which nitric oxide (NO), generated during the early permeability-inducing phase, triggers a delayed cAMP/Epac1 signaling cascade that actively promotes barrier restoration—even in the continued presence of the initiating stimulus. This paradigm shift reframes NO from being solely a permeability-promoting mediator to serving as the initiating signal of a coordinated “inactivation” program (Figure 1).

Once engaged, Epac1–Rap1/Rac1 signaling drives cortical actin polymerization, disassembly of central stress fibers, and reassembly of adherens and tight junction proteins such as VE-cadherin, β-catenin, and ZO-1 at sites of cell–cell contact. Microtubule stabilization via CLIP-170 and IQGAP1 recruitment supports targeted junctional trafficking, while concurrent suppression of RhoA/ROCK activity prevents re-opening of paracellular gaps. A key feature of this repair program is the retro-translocation of endothelial nitric oxide synthase (eNOS) from the cytosol back to caveolar membrane domains, limiting cytosolic NO pools, preventing aberrant S-nitrosylation of junctional proteins, and restoring spatially restricted NO signaling compatible with barrier stability. Epac1 activation also leads to phosphorylation of vasodilator-stimulated phosphoprotein (VASP), reinforcing cortical actin structures and strengthening intercellular adhesion.

This integrated NO/cAMP/Epac1 pathway acts as a master coordinator of cytoskeletal remodeling, junctional reassembly, and focal adhesion turnover. Experimental evidence supports its relevance: in vitro, human microvascular endothelial cells recover from PAF-induced hyperpermeability upon Epac1 activation; in vivo, murine models exhibit a self-limiting permeability response with Epac1-dependent barrier sealing.

The recognition of active functional inactivation has major translational implications. Pharmacological activation of Epac1, selective enhancement of Rap1/Rac1 signaling, or targeted suppression of RhoA/ROCK during the repair phase could accelerate endothelial sealing in acute inflammatory conditions such as sepsis, acute respiratory distress syndrome, and ischemia–reperfusion injury. Furthermore, applying these principles to macrovascular beds may offer new strategies to limit LDL entry and foam cell formation in early atherosclerosis, complementing lipid-lowering and anti-inflammatory therapies.

In summary, the active inactivation of endothelial hyperpermeability represents a fundamental advance in vascular biology—integrating cytoskeletal, junctional, and NO signaling into a unified repair program. Harnessing this pathway offers the potential to restore barrier integrity and improve outcomes across a wide spectrum of acute and chronic vascular diseases.

## Data Availability

The datasets generated and/or analyzed during the current study are available from the corresponding author, M.A.L., upon reasonable request.

## Abbreviations

8cPT-cAMP	8-pCPT-2′-O-Me-cAMP
ARDS	Acute respiratory distress syndrome
cAMP	Cyclic adenosine monophosphate
Cav-1	Caveolin-1
CLIP-170	Cytoplasmic linker protein of 170 kDa
eNOS	Endothelial nitric oxide synthase
Epac1	Exchange protein directly activated by cAMP 1
HMVECs	Human microvascular endothelial cells
sp90	Heat shock protein 90
ICAM-1	Intercellular adhesion molecule-1
IL-1β	Interleukin-1 beta
I/R	Ischemia–reperfusion
KLF2	Krüppel-like factor 2
MLCK	Myosin light chain kinase
NF-κB	Nuclear factor kappa-light-chain-enhancer of activated B cells
NO	Nitric oxide
NOSTRIN	Nitric oxide synthase trafficking inducer
oxLDL	Oxidized low-density lipoprotein
PAF	Platelet-activating factor
PARs	Protease-activated receptors
PKA	Protein kinase A
PKG	Protein kinase G
PP2A	Protein phosphatase 2A
Rap1	Ras-proximate-1 small GTP-binding protein
RhoA	Ras homolog family member A
ROCK	Rho-associated coiled-coil containing protein kinase
ROS	Reactive oxygen species
S-nitrosylation	Covalent attachment of a nitric oxide group to a cysteine thiol
TNF-α	Tumor necrosis factor alpha
VASP	Vasodilator-stimulated phosphoprotein
VCAM-1	Vascular cell adhesion molecule-1
VE-cadherin	Vascular endothelial cadherin
VEGF	Vascular endothelial growth factor
VEGFR-2	Vascular endothelial growth factor receptor-2
ZO-1	Zonula occludens-1

## References

[1] Feletou M. (2011). The Endothelium: Part 1: Multiple Functions of the Endothelial Cells-Focus on Endothelium-Derived Vasoactive Mediators Integrated Systems Physiology: From Molecule to Function to Disease.

[2] Bermejo-Martin J.F., Martin-Fernandez M., Lopez-Mestanza C., Duque P., Almansa R. (2018). Shared Features of Endothelial Dysfunction between Sepsis and Its Preceding Risk Factors (Aging and Chronic Disease). J. Clin. Med..

[3] Wang X., He B. (2024). Endothelial dysfunction: Molecular mechanisms and clinical implications. MedComm.

[4] Aslam M., Gunduz D., Schuler D., Li L., Sharifpanah F., Sedding D., Piper H.M., Noll T. (2011). Intermedin induces loss of coronary microvascular endothelial barrier via derangement of actin cytoskeleton: Role of RhoA and Rac1. Cardiovasc. Res..

[5] Aslam M., Tanislav C., Troidl C., Schulz R., Hamm C., Gunduz D. (2014). cAMP controls the restoration of endothelial barrier function after thrombin-induced hyperpermeability via Rac1 activation. Physiol. Rep..

[6] Wei L., Dankwa S., Vijayan K., Smith J.D., Kaushansky A. (2024). Interrogating endothelial barrier regulation by temporally resolved kinase network generation. Life Sci. Alliance.

[7] Nepali P.R., Burboa P.C., Lillo M.A., Mujica P.E., Iwahashi T., Zhang J., Duran R.G., Boric M., Golenhofen N., Kim D.D. (2023). Endothelial mechanisms for inactivation of inflammation-induced hyperpermeability. Am. J. Physiol. Heart Circ. Physiol..

[8] Gavard J. (2009). Breaking the VE-cadherin bonds. FEBS Lett..

[9] Vestweber D. (2015). How leukocytes cross the vascular endothelium. Nat. Rev. Immunol..

[10] Komarova Y., Malik A.B. (2010). Regulation of endothelial permeability via paracellular and transcellular transport pathways. Annu. Rev. Physiol..

[11] Orfanos S.E., Mavrommati I., Korovesi I., Roussos C. (2004). Pulmonary endothelium in acute lung injury: From basic science to the critically ill. Intensive Care Med..

[12] Carman C.V., Springer T.A. (2004). A transmigratory cup in leukocyte diapedesis both through individual vascular endothelial cells and between them. J. Cell Biol..

[13] Korayem A.H., Mujica P.E., Aramoto H., Duran R.G., Nepali P.R., Kim D.D., Harris A.L., Sanchez F.A., Duran W.N. (2017). Endothelial cAMP deactivates ischemia-reperfusion-induced microvascular hyperpermeability via Rap1-mediated mechanisms. Am. J. Physiol. Heart Circ. Physiol..

[14] Duran W.N., Beuve A.V., Sanchez F.A. (2013). Nitric oxide, S-nitrosation, and endothelial permeability. IUBMB Life.

[15] Duran W.N., Breslin J.W., Sanchez F.A. (2010). The NO cascade, eNOS location, and microvascular permeability. Cardiovasc. Res..

[16] Sanchez F.A., Kim D.D., Duran R.G., Meininger C.J., Duran W.N. (2008). Internalization of eNOS via caveolae regulates PAF-induced inflammatory hyperpermeability to macromolecules. Am. J. Physiol. Heart Circ. Physiol..

[17] Sanchez F.A., Rana R., Gonzalez F.G., Iwahashi T., Duran R.G., Fulton D.J., Beuve A.V., Kim D.D., Duran W.N. (2011). Functional significance of cytosolic endothelial nitric-oxide synthase (eNOS): Regulation of hyperpermeability. J. Biol. Chem..

[18] Tzima E. (2006). Role of small GTPases in endothelial cytoskeletal dynamics and the shear stress response. Circ. Res..

[19] Zebda N., Tian Y., Tian X., Gawlak G., Higginbotham K., Reynolds A.B., Birukova A.A., Birukov K.G. (2013). Interaction of p190RhoGAP with C-terminal domain of p120-catenin modulates endothelial cytoskeleton and permeability. J. Biol. Chem..

[20] Gavard J., Gutkind J.S. (2008). Protein kinase C-related kinase and ROCK are required for thrombin-induced endothelial cell permeability downstream from Galpha12/13 and Galpha11/q. J. Biol. Chem..

[21] Sanchez F.A., Rana R., Kim D.D., Iwahashi T., Zheng R., Lal B.K., Gordon D.M., Meininger C.J., Duran W.N. (2009). Internalization of eNOS and NO delivery to subcellular targets determine agonist-induced hyperpermeability. Proc. Natl. Acad. Sci. USA.

[22] Di Lorenzo A., Lin M.I., Murata T., Landskroner-Eiger S., Schleicher M., Kothiya M., Iwakiri Y., Yu J., Huang P.L., Sessa W.C. (2013). eNOS-derived nitric oxide regulates endothelial barrier function through VE-cadherin and Rho GTPases. J. Cell Sci..

[23] Fulton D., Fontana J., Sowa G., Gratton J.P., Lin M., Li K.X., Michell B., Kemp B.E., Rodman D., Sessa W.C. (2002). Localization of endothelial nitric-oxide synthase phosphorylated on serine 1179 and nitric oxide in Golgi and plasma membrane defines the existence of two pools of active enzyme. J. Biol. Chem..

[24] Garcia-Ponce A., Schuster K., Doskeland S.O., Reed R.K., Curry F.E., Waschke J., Radeva M.Y. (2020). Epac1 Is Crucial for Maintenance of Endothelial Barrier Function through A Mechanism Partly Independent of Rac1. Cells.

[25] Birukova A.A., Burdette D., Moldobaeva N., Xing J., Fu P., Birukov K.G. (2010). Rac GTPase is a hub for protein kinase A and Epac signaling in endothelial barrier protection by cAMP. Microvasc. Res..

[26] Kooistra M.R., Corada M., Dejana E., Bos J.L. (2005). Epac1 regulates integrity of endothelial cell junctions through VE-cadherin. FEBS Lett..

[27] Schlegel N., Waschke J. (2009). VASP is involved in cAMP-mediated Rac 1 activation in microvascular endothelial cells. Am. J. Physiol. Cell Physiol..

[28] Ni Y., Teng T., Li R., Simonyi A., Sun G.Y., Lee J.C. (2017). TNFalpha alters occludin and cerebral endothelial permeability: Role of p38MAPK. PLoS ONE.

[29] Jones J.H., Minshall R.D. (2022). Endothelial Transcytosis in Acute Lung Injury: Emerging Mechanisms and Therapeutic Approaches. Front. Physiol..

[30] Versele R., Sevin E., Gosselet F., Fenart L., Candela P. (2022). TNF-alpha and IL-1beta Modulate Blood-Brain Barrier Permeability and Decrease Amyloid-beta Peptide Efflux in a Human Blood-Brain Barrier Model. Int. J. Mol. Sci..

[31] Yue Q., Leng X., Xie N., Zhang Z., Yang D., Hoi M.P.M. (2024). Endothelial Dysfunctions in Blood-Brain Barrier Breakdown in Alzheimer’s Disease: From Mechanisms to Potential Therapies. CNS Neurosci. Ther..

[32] Vielmuth F., Radeva M.Y., Yeruva S., Sigmund A.M., Waschke J. (2023). cAMP: A master regulator of cadherin-mediated binding in endothelium, epithelium and myocardium. Acta Physiol..

[33] Adderley S.P., Zhang X.E., Breslin J.W. (2015). Involvement of the H1 Histamine Receptor, p38 MAP Kinase, Myosin Light Chains Kinase, and Rho/ROCK in Histamine-Induced Endothelial Barrier Dysfunction. Microcirculation.

[34] Wojciak-Stothard B., Potempa S., Eichholtz T., Ridley A.J. (2001). Rho and Rac but not Cdc42 regulate endothelial cell permeability. J. Cell Sci..

[35] van Nieuw Amerongen G.P., van Delft S., Vermeer M.A., Collard J.G., van Hinsbergh V.W. (2000). Activation of RhoA by thrombin in endothelial hyperpermeability: Role of Rho kinase and protein tyrosine kinases. Circ. Res..

[36] Gupta G.S., Sharma P.K. (1995). Molecular inactivation of testicular hyaluronidase in solid state after proton irradiation: A study based on target size, substrate binding and thermodynamic analysis of heat denaturation. Indian J. Biochem. Biophys..

[37] Giri H., Srivastava A.K., Naik U.P. (2022). Apoptosis signal-regulating kinase-1 regulates thrombin-induced endothelial permeability. Vasc. Pharmacol..

[38] Kugelmann D., Rotkopf L.T., Radeva M.Y., Garcia-Ponce A., Walter E., Waschke J. (2018). Histamine causes endothelial barrier disruption via Ca^2+^-mediated RhoA activation and tension at adherens junctions. Sci. Rep..

[39] Shen Q., Rigor R.R., Pivetti C.D., Wu M.H., Yuan S.Y. (2010). Myosin light chain kinase in microvascular endothelial barrier function. Cardiovasc. Res..

[40] Li Z., Yin M., Zhang H., Ni W., Pierce R.W., Zhou H.J., Min W. (2020). BMX Represses Thrombin-PAR1-Mediated Endothelial Permeability and Vascular Leakage During Early Sepsis. Circ. Res..

[41] Grimsey N.J., Trejo J. (2016). Integration of endothelial protease-activated receptor-1 inflammatory signaling by ubiquitin. Curr. Opin. Hematol..

[42] Bae J.S., Kim Y.U., Park M.K., Rezaie A.R. (2009). Concentration dependent dual effect of thrombin in endothelial cells via Par-1 and Pi3 Kinase. J. Cell Physiol..

[43] Minjares M., Wu W., Wang J.M. (2023). Oxidative Stress and MicroRNAs in Endothelial Cells under Metabolic Disorders. Cells.

[44] Munzel T., Camici G.G., Maack C., Bonetti N.R., Fuster V., Kovacic J.C. (2017). Impact of Oxidative Stress on the Heart and Vasculature: Part 2 of a 3-Part Series. J. Am. Coll. Cardiol..

[45] Szabo C., Modis K. (2010). Pathophysiological roles of peroxynitrite in circulatory shock. Shock.

[46] Prolo C., Piacenza L., Radi R. (2024). Peroxynitrite: A multifaceted oxidizing and nitrating metabolite. Curr. Opin. Chem. Biol..

[47] Walter F.R., Harazin A., Toth A.E., Veszelka S., Santa-Maria A.R., Barna L., Kincses A., Biczo G., Balla Z., Kui B. (2022). Blood-brain barrier dysfunction in L-ornithine induced acute pancreatitis in rats and the direct effect of L-ornithine on cultured brain endothelial cells. Fluids Barriers CNS.

[48] Chiba H., Osanai M., Murata M., Kojima T., Sawada N. (2008). Transmembrane proteins of tight junctions. Biochim. Biophys. Acta.

[49] Forstermann U., Sessa W.C. (2012). Nitric oxide synthases: Regulation and function. Eur. Heart J..

[50] Bedard K., Krause K.H. (2007). The NOX family of ROS-generating NADPH oxidases: Physiology and pathophysiology. Physiol. Rev..

[51] Brandes R.P., Weissmann N., Schroder K. (2014). Nox family NADPH oxidases: Molecular mechanisms of activation. Free Radic. Biol. Med..

[52] Radi R. (2018). Oxygen radicals, nitric oxide, and peroxynitrite: Redox pathways in molecular medicine. Proc. Natl. Acad. Sci. USA.

[53] Pacher P., Beckman J.S., Liaudet L. (2007). Nitric oxide and peroxynitrite in health and disease. Physiol. Rev..

[54] Rao R. (2009). Occludin phosphorylation in regulation of epithelial tight junctions. Ann. N. Y. Acad. Sci..

[55] Rao R. (2008). Oxidative stress-induced disruption of epithelial and endothelial tight junctions. Front. Biosci..

[56] Haorah J., Heilman D., Knipe B., Chrastil J., Leibhart J., Ghorpade A., Miller D.W., Persidsky Y. (2005). Ethanol-induced activation of myosin light chain kinase leads to dysfunction of tight junctions and blood-brain barrier compromise. Alcohol. Clin. Exp. Res..

[57] Konior A., Schramm A., Czesnikiewicz-Guzik M., Guzik T.J. (2014). NADPH oxidases in vascular pathology. Antioxid. Redox Signal..

[58] Karki P., Birukov K.G. (2019). Rho and Reactive Oxygen Species at Crossroads of Endothelial Permeability and Inflammation. Antioxid. Redox Signal..

[59] Zhou H., Sun Y., Zhang L., Kang W., Li N., Li Y. (2018). The RhoA/ROCK pathway mediates high glucose-induced cardiomyocyte apoptosis via oxidative stress, JNK, and p38MAPK pathways. Diabetes Metab. Res. Rev..

[60] Bowie A., O’Neill L.A. (2000). Oxidative stress and nuclear factor-kappaB activation: A reassessment of the evidence in the light of recent discoveries. Biochem. Pharmacol..

[61] Zhang T., Ma C., Zhang Z., Zhang H., Hu H. (2021). NF-kappaB signaling in inflammation and cancer. MedComm.

[62] Tian Y., Gawlak G., O’Donnell J.J., Birukova A.A., Birukov K.G. (2016). Activation of Vascular Endothelial Growth Factor (VEGF) Receptor 2 Mediates Endothelial Permeability Caused by Cyclic Stretch. J. Biol. Chem..

[63] Soares S.R., Gomez R., Simon C., Garcia-Velasco J.A., Pellicer A. (2008). Targeting the vascular endothelial growth factor system to prevent ovarian hyperstimulation syndrome. Hum. Reprod. Update.

[64] Sriram K., Laughlin J.G., Rangamani P., Tartakovsky D.M. (2016). Shear-Induced Nitric Oxide Production by Endothelial Cells. Biophys. J..

[65] Gavard J., Gutkind J.S. (2008). VE-cadherin and claudin-5: It takes two to tango. Nat. Cell Biol..

[66] Eliceiri B.P., Paul R., Schwartzberg P.L., Hood J.D., Leng J., Cheresh D.A. (1999). Selective requirement for Src kinases during VEGF-induced angiogenesis and vascular permeability. Mol. Cell.

[67] Gavard J., Gutkind J.S. (2006). VEGF controls endothelial-cell permeability by promoting the beta-arrestin-dependent endocytosis of VE-cadherin. Nat. Cell Biol..

[68] Li X., Padhan N., Sjostrom E.O., Roche F.P., Testini C., Honkura N., Sainz-Jaspeado M., Gordon E., Bentley K., Philippides A. (2016). VEGFR2 pY949 signalling regulates adherens junction integrity and metastatic spread. Nat. Commun..

[69] Smith R.O., Ninchoji T., Gordon E., Andre H., Dejana E., Vestweber D., Kvanta A., Claesson-Welsh L. (2020). Vascular permeability in retinopathy is regulated by VEGFR2 Y949 signaling to VE-cadherin. eLife.

[70] Zhou W., Liu K., Zeng L., He J., Gao X., Gu X., Chen X., Jing Li J., Wang M., Wu D. (2022). Targeting VEGF-A/VEGFR2 Y949 Signaling-Mediated Vascular Permeability Alleviates Hypoxic Pulmonary Hypertension. Circulation.

[71] Dimmeler S., Dernbach E., Zeiher A.M. (2000). Phosphorylation of the endothelial nitric oxide synthase at ser-1177 is required for VEGF-induced endothelial cell migration. FEBS Lett..

[72] Michell B.J., Griffiths J.E., Mitchelhill K.I., Rodriguez-Crespo I., Tiganis T., Bozinovski S., de Montellano P.R., Kemp B.E., Pearson R.B. (1999). The Akt kinase signals directly to endothelial nitric oxide synthase. Curr. Biol..

[73] Dvorak A.M., Feng D. (2001). The vesiculo-vacuolar organelle (VVO). A new endothelial cell permeability organelle. J. Histochem. Cytochem..

[74] Argaw A.T., Gurfein B.T., Zhang Y., Zameer A., John G.R. (2009). VEGF-mediated disruption of endothelial CLN-5 promotes blood-brain barrier breakdown. Proc. Natl. Acad. Sci. USA.

[75] Greene C., Campbell M. (2016). Tight junction modulation of the blood brain barrier: CNS delivery of small molecules. Tissue Barriers.

[76] Kim Y.M., Kim S.J., Tatsunami R., Yamamura H., Fukai T., Ushio-Fukai M. (2017). ROS-induced ROS release orchestrated by Nox4, Nox2, and mitochondria in VEGF signaling and angiogenesis. Am. J. Physiol. Cell Physiol..

[77] Ushio-Fukai M. (2007). VEGF signaling through NADPH oxidase-derived ROS. Antioxid. Redox Signal..

[78] Evangelista A.M., Thompson M.D., Bolotina V.M., Tong X., Cohen R.A. (2012). Nox4- and Nox2-dependent oxidant production is required for VEGF-induced SERCA cysteine-674 S-glutathiolation and endothelial cell migration. Free Radic. Biol. Med..

[79] Feron O., Michel J.B., Sase K., Michel T. (1998). Dynamic regulation of endothelial nitric oxide synthase: Complementary roles of dual acylation and caveolin interactions. Biochemistry.

[80] Garcia-Cardena G., Martasek P., Masters B.S., Skidd P.M., Couet J., Li S., Lisanti M.P., Sessa W.C. (1997). Dissecting the interaction between nitric oxide synthase (NOS) and caveolin. Functional significance of the nos caveolin binding domain in vivo. J. Biol. Chem..

[81] Fulton D., Gratton J.P., Sessa W.C. (2001). Post-translational control of endothelial nitric oxide synthase: Why isn’t calcium/calmodulin enough?. J. Pharmacol. Exp. Ther..

[82] Liu J., Hughes T.E., Sessa W.C. (1997). The first 35 amino acids and fatty acylation sites determine the molecular targeting of endothelial nitric oxide synthase into the Golgi region of cells: A green fluorescent protein study. J. Cell Biol..

[83] Fulton D., Babbitt R., Zoellner S., Fontana J., Acevedo L., McCabe T.J., Iwakiri Y., Sessa W.C. (2004). Targeting of endothelial nitric-oxide synthase to the cytoplasmic face of the Golgi complex or plasma membrane regulates Akt- versus calcium-dependent mechanisms for nitric oxide release. J. Biol. Chem..

[84] Marin N., Zamorano P., Carrasco R., Mujica P., Gonzalez F.G., Quezada C., Meininger C.J., Boric M.P., Duran W.N., Sanchez F.A. (2012). S-Nitrosation of beta-catenin and p120 catenin: A novel regulatory mechanism in endothelial hyperpermeability. Circ. Res..

[85] Guequen A., Carrasco R., Zamorano P., Rebolledo L., Burboa P., Sarmiento J., Boric M.P., Korayem A., Duran W.N., Sanchez F.A. (2016). S-nitrosylation regulates VE-cadherin phosphorylation and internalization in microvascular permeability. Am. J. Physiol. Heart Circ. Physiol..

[86] Fulton D., Gratton J.P., McCabe T.J., Fontana J., Fujio Y., Walsh K., Franke T.F., Papapetropoulos A., Sessa W.C. (1999). Regulation of endothelium-derived nitric oxide production by the protein kinase Akt. Nature.

[87] Fernandez-Hernando C., Fukata M., Bernatchez P.N., Fukata Y., Lin M.I., Bredt D.S., Sessa W.C. (2006). Identification of Golgi-localized acyl transferases that palmitoylate and regulate endothelial nitric oxide synthase. J. Cell Biol..

[88] Iwakiri Y. (2011). S-nitrosylation of proteins: A new insight into endothelial cell function regulated by eNOS-derived NO. Nitric Oxide.

[89] Chakraborty S., Ain R. (2017). Nitric-oxide synthase trafficking inducer is a pleiotropic regulator of endothelial cell function and signaling. J. Biol. Chem..

[90] Garcia-Cardena G., Fan R., Shah V., Sorrentino R., Cirino G., Papapetropoulos A., Sessa W.C. (1998). Dynamic activation of endothelial nitric oxide synthase by Hsp90. Nature.

[91] Chen J.X., Meyrick B. (2004). Hypoxia increases Hsp90 binding to eNOS via PI3K-Akt in porcine coronary artery endothelium. Lab. Investig..

[92] Pastorekova S., Parkkila S., Pastorek J., Supuran C.T. (2004). Carbonic anhydrases: Current state of the art, therapeutic applications and future prospects. J. Enzym. Inhib. Med. Chem..

[93] Ghosh S., Gachhui R., Crooks C., Wu C., Lisanti M.P., Stuehr D.J. (1998). Interaction between caveolin-1 and the reductase domain of endothelial nitric-oxide synthase. Consequences for catalysis. J. Biol. Chem..

[94] Rampersad S.N., Freitag S.I., Hubert F., Brzezinska P., Butler N., Umana M.B., Wudwud A.R., Maurice D.H. (2016). EPAC1 promotes adaptive responses in human arterial endothelial cells subjected to low levels of laminar fluid shear stress: Implications in flow-related endothelial dysfunction. Cell. Signal..

[95] Belvitch P., Htwe Y.M., Brown M.E., Dudek S. (2018). Cortical Actin Dynamics in Endothelial Permeability. Curr. Top. Membr..

[96] Vogel S.M., Malik A.B. (2012). Cytoskeletal dynamics and lung fluid balance. Compr. Physiol..

[97] Wentworth J.K., Pula G., Poole A.W. (2006). Vasodilator-stimulated phosphoprotein (VASP) is phosphorylated on Ser157 by protein kinase C-dependent and -independent mechanisms in thrombin-stimulated human platelets. Biochem. J..

[98] Arthur A.L., Crawford A., Houdusse A., Titus M.A. (2021). VASP-mediated actin dynamics activate and recruit a filopodia myosin. eLife.

[99] Noda K., Zhang J., Fukuhara S., Kunimoto S., Yoshimura M., Mochizuki N. (2010). Vascular endothelial-cadherin stabilizes at cell-cell junctions by anchoring to circumferential actin bundles through alpha- and beta-catenins in cyclic AMP-Epac-Rap1 signal-activated endothelial cells. Mol. Biol. Cell.

[100] Kris A.S., Kamm R.D., Sieminski A.L. (2008). VASP involvement in force-mediated adherens junction strengthening. Biochem. Biophys. Res. Commun..

[101] Gratton J.P., Fontana J., O’Connor D.S., Garcia-Cardena G., McCabe T.J., Sessa W.C. (2000). Reconstitution of an endothelial nitric-oxide synthase (eNOS), hsp90, and caveolin-1 complex in vitro. Evidence that hsp90 facilitates calmodulin stimulated displacement of eNOS from caveolin-1. J. Biol. Chem..

[102] Moztarzadeh S., Sepic S., Hamad I., Waschke J., Radeva M.Y., Garcia-Ponce A. (2024). Cortactin is in a complex with VE-cadherin and is required for endothelial adherens junction stability through Rap1/Rac1 activation. Sci. Rep..

[103] Birukova A.A., Tian X., Tian Y., Higginbotham K., Birukov K.G. (2013). Rap-afadin axis in control of Rho signaling and endothelial barrier recovery. Mol. Biol. Cell.

[104] Pannekoek W.J., Post A., Bos J.L. (2014). Rap1 signaling in endothelial barrier control. Cell Adhes. Migr..

[105] Parinandi N., Gerasimovskaya E., Verin A. (2022). Editorial: Molecular mechanisms of lung endothelial permeability. Front. Physiol..

[106] Huang R.T., Wu D., Meliton A., Oh M.J., Krause M., Lloyd J.A., Nigdelioglu R., Hamanaka R.B., Jain M.K., Birukova A. (2017). Experimental Lung Injury Reduces Kruppel-like Factor 2 to Increase Endothelial Permeability via Regulation of RAPGEF3-Rac1 Signaling. Am. J. Respir. Crit. Care Med..

[107] Fledderus J.O., Boon R.A., Volger O.L., Hurttila H., Yla-Herttuala S., Pannekoek H., Levonen A.L., Horrevoets A.J. (2008). KLF2 primes the antioxidant transcription factor Nrf2 for activation in endothelial cells. Arterioscler. Thromb. Vasc. Biol..

[108] Cahill P.A., Redmond E.M. (2016). Vascular endothelium—Gatekeeper of vessel health. Atherosclerosis.

[109] Mundi S., Massaro M., Scoditti E., Carluccio M.A., van Hinsbergh V.W.M., Iruela-Arispe M.L., De Caterina R. (2018). Endothelial permeability, LDL deposition, and cardiovascular risk factors-a review. Cardiovasc. Res..

[110] Jiang H., Zhou Y., Nabavi S.M., Sahebkar A., Little P.J., Xu S., Weng J., Ge J. (2022). Mechanisms of Oxidized LDL-Mediated Endothelial Dysfunction and Its Consequences for the Development of Atherosclerosis. Front. Cardiovasc. Med..

[111] Libby P., Buring J.E., Badimon L., Hansson G.K., Deanfield J., Bittencourt M.S., Tokgozoglu L., Lewis E.F. (2019). Atherosclerosis. Nat. Rev. Dis. Primers.

[112] Gimbrone M.A., Garcia-Cardena G. (2016). Endothelial Cell Dysfunction and the Pathobiology of Atherosclerosis. Circ. Res..

[113] Moore K.J., Sheedy F.J., Fisher E.A. (2013). Macrophages in atherosclerosis: A dynamic balance. Nat. Rev. Immunol..

[114] Abbott N.J., Patabendige A.A., Dolman D.E., Yusof S.R., Begley D.J. (2010). Structure and function of the blood-brain barrier. Neurobiol. Dis..

[115] Ben-Zvi A., Lacoste B., Kur E., Andreone B.J., Mayshar Y., Yan H., Gu C. (2014). Mfsd2a is critical for the formation and function of the blood-brain barrier. Nature.

[116] Curry F.E., Adamson R.H. (2012). Endothelial glycocalyx: Permeability barrier and mechanosensor. Ann. Biomed. Eng..

[117] Daneman R., Prat A. (2015). The blood-brain barrier. Cold Spring Harb. Perspect. Biol..

[118] Satchell S.C., Braet F. (2009). Glomerular endothelial cell fenestrations: An integral component of the glomerular filtration barrier. Am. J. Physiol. Ren. Physiol..

[119] Kolarova H., Ambruzova B., Svihalkova Sindlerova L., Klinke A., Kubala L. (2014). Modulation of endothelial glycocalyx structure under inflammatory conditions. Mediat. Inflamm..

[120] Salmon A.H., Satchell S.C. (2012). Endothelial glycocalyx dysfunction in disease: Albuminuria and increased microvascular permeability. J. Pathol..

[121] Jeansson M., Gawlik A., Anderson G., Li C., Kerjaschki D., Henkelman M., Quaggin S.E. (2011). Angiopoietin-1 is essential in mouse vasculature during development and in response to injury. J. Clin. Investig..

